# Triclocarban and Triclosan Inhibit Human Aromatase via Different Mechanisms

**DOI:** 10.1155/2017/8284097

**Published:** 2017-12-10

**Authors:** Huitao Li, Yu Zhao, Lanlan Chen, Ying Su, Xiaoheng Li, Lixu Jin, Ren-Shan Ge

**Affiliations:** ^1^Center of Scientific Research, The Second Affiliated Hospital and Yuying Children's Hospital of Wenzhou Medical University, Wenzhou, Zhejiang 325027, China; ^2^The Gynecology and Obstetrics Department, The Second Affiliated Hospital and Yuying Children's Hospital of Wenzhou Medical University, Wenzhou, Zhejiang 325027, China; ^3^The Gynecology and Obstetrics Department, The First Affiliated Hospital of Wenzhou Medical University, Wenzhou, Zhejiang 325000, China

## Abstract

Human aromatase (CYP19A1) is an important enzyme, which produces estrogen from androgen for maintaining the female reproductive function and pregnancy. Triclocarban and triclosan are antimicrobial chemicals added to personal care, household, and industrial products. They could be endocrine disruptors and may disrupt human CYP19A1 activity. In the present study, we investigated the effects of triclocarban and triclosan on estradiol production and human CYP19A1 activity in JEG-3 cells. Triclocarban and triclosan reduced estradiol production in JEG-3 cells. Triclocarban and triclosan inhibited human CYP19A1 with IC_50_ values of 15.81 and 6.26 *μ*M, respectively. Triclosan competitively inhibited CYP19A1, while triclocarban noncompetitively inhibited this enzyme. Docking study showed that triclosan bound to the steroid-binding pocket of CYP19A1, while triclocarban was off this target, suggesting a different mechanism. In conclusion, triclocarban and triclosan are inhibitors of human CYP19A1.

## 1. Introduction

**Figure 1:**
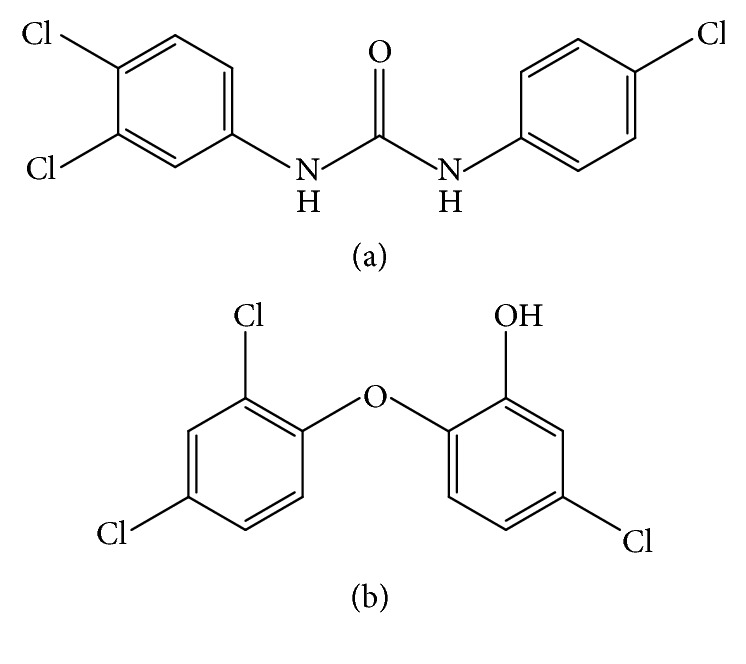
Structures of triclocarban and triclosan. (a) Triclocarban; (b) triclosan.

Triclosan and triclocarban have been used as antimicrobial chemicals in many personal care, household, and industrial products for several decades. Structurally, triclosan (Figure 1) has similarities with diethylstilbestrol and bisphenol A, which are possible endocrine disruptors [1–3]. Triclocarban has also similar chemical properties to triclosan (Figure 1). Because they are produced in a large quantity, they are absorbed into the human body of the general population. Majority (75%) of the urine samples from US population in a recent survey had 0.0024–3.790 mg/L of triclosan and its metabolites [4]. Triclocarban and its metabolites were also detected to be 100 ng/L in 36.9% of the urine samples in US adults [5].

Both triclosan and triclocarban are classified to be the endocrine disruptors. Triclosan and triclocarban have been shown to exert either estrogenic or antiestrogenic activity in different model systems [6]. This difference could be from their multiple toxicological properties. In one study, very low concentrations of triclosan stimulated proliferation of breast cancer cells possibly via directly binding to estrogen receptors [7]. However, in the presence of low concentrations of estradiol, a nature potent estrogen, triclosan behaved as antiestrogenic, possibly via different mechanisms [7]. Triclosan also disrupts placental functions. In a rodent model, triclosan inhibited steroid hormone (including estradiol) production in rat placenta [8]. Triclosan was also found to inhibit another estrogen metabolic enzyme, estrogen sulfotransferase, in both human and rodents [9]. In this regard, both triclosan and triclocarban could interfere with estradiol production.

**Figure 2:**
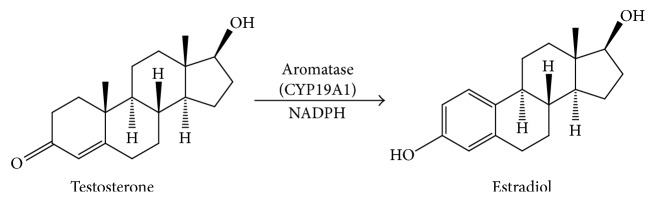
Scheme of human aromatase (CYP19A1) catalytic reaction. CYP19A1 catalyzes testosterone into estradiol.

One organ for estradiol production is human placenta. Human placenta contains aromatase (CYP19A1). CYP19A1 is a cytochrome P450 enzyme. This enzyme is a complex, including NADPH, cytochrome P450 reductase, and heme, catalyzing three steps of hydroxylation, converting testosterone to estradiol (Figure 2).

Placental estradiol is very critical to promote placental growth and stimulates the blood flow to provide the optimal exchange of gases and nutrients [10]. In the present study, we investigated the effects of triclosan and triclocarban on this enzyme.

## 2. Materials and Methods

### 2.1. Materials

Testosterone, dehydroepiandrosterone (DHEA), and estradiol were purchased from Steraloids (Newport, RI). Triclocarban and triclosan were purchased from Sigma-Aldrich (St. Louis, MO). Triclocarban and triclosan were dissolved in DMSO for assay. JEG-3 cells were obtained from ATCC (Manassas, VA) for estradiol production assay because they contain CYP19A1.

### 2.2. JEG-3 Cell Culture and Treatment

In the present study, JEG-3 cell has been widely used as a model for placental steroid production analysis [11, 12]. This cell line was derived from a human choriocarcinoma, and it retained the higher capacity to produce estradiol when an exogenous DHEA was provided [13]. JEG-3 cells secreted estrone (1 ± 0.16 ng/ml) and estradiol (11 ± 3.1 ng/ml) in the presence of excess DHEA [14]. The JEG-3 cell culture and DHEA treatment were performed as previously described [15]. JEG-3 cells were seeded at 10^5^ cells per well in 24-well plates and cultured in MEM medium with phenol red containing 10% fetal calf serum at 37°C and 5% CO2 in the presence of various concentrations of triclocarban or triclosan with 10 *μ*M DHEA (for estradiol assay) for 12 h. For cytotoxicity analysis, cells were treated with 100 *μ*M triclosan or triclocarban and the cells were tested for their viability as discussed below. JEG-3 cells were also treated with different concentrations of triclocarban or triclosan for 12 h. Media were collected for the measurement of estradiol levels.

### 2.3. Cell Viability Assay

JEG-3 cells were seeded at 5,000 cells per well in 100 *μ*l of medium in 96-well plates and the cells were treated with 100 *μ*M of triclocarban or triclosan. For the cell viability assay, the Cell Counting Kit-8 (Sigma-Aldrich, MO) was used according to the manufacturer's instructions. The kit allows convenient assays by utilizing highly water-soluble tetrazolium salt to produce a water-soluble formazan dye upon reduction in the presence of an electron mediator. CCK-8 solution (10 *μ*l) was pipetted into each well, and cell mixture was incubated for 2 h at 37°C. Then, the plate was read by a microplate reader at 450 nm (OD450). Five samples per point were measured and averaged.

### 2.4. Preparation of JEG-3 Cell Microsomes

JEG-3 cell microsomes were prepared as previously described [16]. Briefly, JEG-3 cells were scraped and homogenized in ice-cold phosphate-buffered saline (0.01 mM, pH 7.2) containing 0.25 mM sucrose, and cell homogenates were centrifuged at 700 ×g for 30 min at 4°C to remove the larger cellular debris. The supernatants were transferred into new tubes and were centrifuged at 10,000 ×g for 30 minutes at 4°C to remove the mitochondria. The resulting supernatants were centrifuged twice at 105,000 ×g for 1 h at 4°C to collect microsomal pellets. Pellets were washed with PBS and resuspended by homogenization.

### 2.5. Measurement of Protein Concentration

The total protein concentrations of JEG-3 cell microsomes were measured by Bio-Rad Protein Assay Kit (cat# 500-0006, Bio-Rad, Hercules, CA) according to manufacturer's protocol. The bovine serum albumin was used as the protein standard. The protein concentrations were adjusted to 4 mg/ml. JEG-3 cell microsome was used to measure CYP19A1 activity.

### 2.6. Measurement of CYP19A1 Activity

CYP19A1 activity in JEG-3 microsomes was measured as previously described [17]. Briefly, CYP19A1 activity mixture contained 100 nM testosterone (about 2 × *K*_m_) and 0.2 mM NADPH in 250 *μ*l PBS and microsomes. The tested chemicals were dissolved in DMSO, and the final DMSO concentration in the enzyme mixture was 0.5%. This concentration of DMSO did not affect the CYP19A1 activity [17]. The time-course and dose range of CYP19A1-containing microsomes were determined. CYP19A1-containing microsomes had a linear enzyme reaction within 30 min. The 30-minute reaction was initiated by addition of 10 *μ*g microsome proteins in presence of different concentrations of triclocarban or triclosan to determine half maximal inhibitory concentration (IC_50_). In order to determine the mode of action for triclocarban or triclosan, different concentrations of testosterone (15.625, 31.25, 62.5, 125, 250, and 500 nM) plus 0.2 mM NADPH were added to the reaction mixture (PBS buffer) containing 10 *μ*g microsome plus triclocarban or triclosan. The reactions were stopped by 10 *μ*l hydrochloric acid (1 N). The estradiol level in the medium was measured by radioimmunoassay to calculate the activity of CYP19A1.

### 2.7. Estradiol Radioimmunoassay

Estradiol levels in the medium were determined in duplicate without prior extraction described [18]. Standard curves of estradiol (300 *μ*L) ranging from 10 to 2000 pg/mL were prepared in triplicate and charcoal-dextran suspension was used to separate the bound and free steroid. The minimum detectable dose of the assay was less than 7.5 pg/mL, and the mean intra-assay coefficient of variation was less than 10%.

### 2.8. Preparation of Protein and Ligand Structures and Docking

The crystal structure data of recombinant human CYP19A1, which has a complex with heme and 4-androstene-3,17-dione, was obtained from PDB [PDB ID: 4KQ8 [19]]. 4KQ8 was used as a docking target for steroid substrate androstenedione as well as triclocarban or triclosan. The structures of 4-androstene-3,17-dione, triclocarban, and triclosan were selected from PubChem (https://pubchem.ncbi.nlm.nih.gov) as ligands for docking. Docking calculations were performed with SwissDock, a docking algorithm based on the docking software EADock DSS [20]. The algorithm consists of a large number of binding modes (typically from 5,000 to 15,000) that are ranked with the most favorable energies with the solvent effect being taken into account using the FACTS implicit solvation model and clustered. The docked file was generated and visualized using the program Chimera 1.1.1 (San Francisco, CA) and the free energy was obtained.

### 2.9. Statistics

Assays were repeated four times. The IC_50_ value was calculated using GraphPad Prism version 6.0 (GraphPad Software Inc., San Diego, CA) using nonlinear regression of curve fit with one-site competition. Lineweaver-Burk plot was selected to determine the mode of action. Data were subjected to analysis by one-way ANOVA followed by ad hoc Turkey's multiple comparison test to identify significant differences between groups when three and more groups were calculated. All data are expressed as means ± SEM. Differences were regarded as significant at *p* < 0.05.

## 3. Results

### 3.1. Effects of Triclosan and Triclocarban on DHEA-Stimulated Estradiol Production

**Figure 3:**
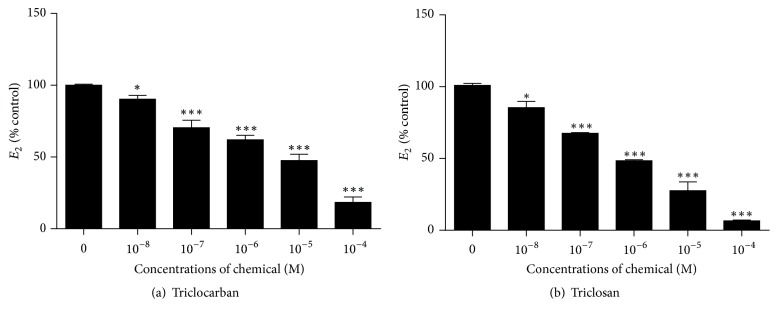
Effects of triclocarban and triclosan on estradiol production in JEG-3 cells. 10 *μ*M DHEA was added for the maximum production of estradiol in JEG-3 cells. Various concentrations (10^−8^ –10^−4^ M) of triclocarban and triclosan were used. Mean ± SEM, *n* = 4. *∗* and *∗∗∗* indicate significant difference when compared to control (0) at *p* ≤ 0.05 and 0.001, respectively.

The tested concentrations (100 *μ*M) of triclocarban and triclosan did not affect the viability of JEG-3 cells (data not shown). Estradiol formation in JEG-3 cells uses exogenous DHEA. When 10 *μ*M DHEA was added, we found that triclocarban and triclosan significantly reduced estradiol production, starting at 100 nM for both triclocarban and triclosan (Figure 3). This indicates that triclocarban and triclosan are able to inhibit CYP19A1 to reduce estradiol production.

### 3.2. Effects of Triclocarban and Triclosan on CYP19A1 Activity

**Figure 4:**
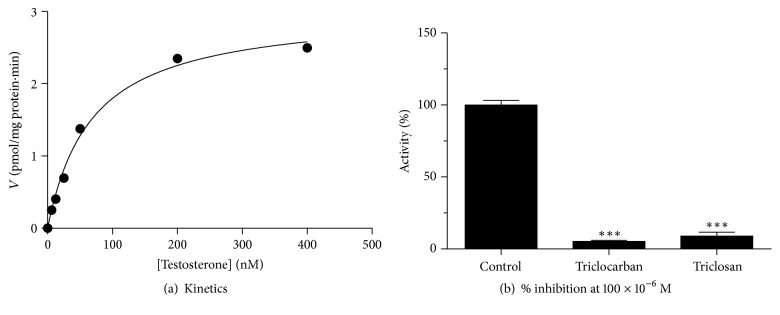
Kinetics of CYP19A1 and effects of triclocarban and triclosan on CYP19A1 activity in JEG-3 microsomes. (a) Kinetics of CYP19A1 in the presence of a range of concentrations of testosterone. (b) Single concentration (100 *μ*M) of triclocarban and triclosan was used to inhibit CYP19A1. Mean ± SEM, *n* = 6. ∗∗∗ indicates a significant difference when compared to control at *p* ≤ 0.001.

CYP19A1 catalyzes the conversion of testosterone to estradiol in JEG-3 cell microsomes. CYP19A1 has *K*_m_ of 68 *μ*M for testosterone and* V*max of 3.020 pmol/mg·min (Figure 4(a) and Table 1).

**Figure 5:**
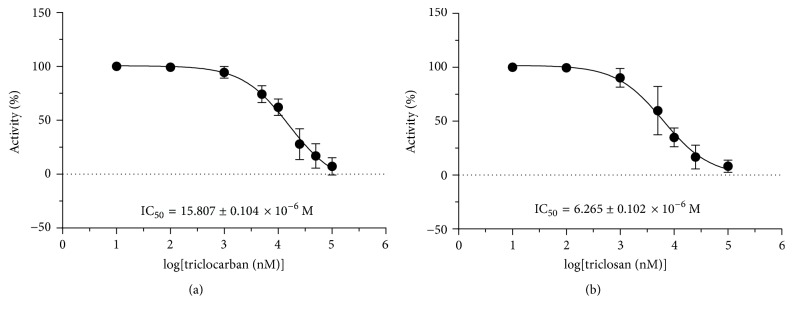
Concentration-dependent inhibition of triclocarban and triclosan on human CYP19A1 activity. Mean ± SD, *n* = 6. (a) Triclocarban; (b) triclosan.

At 100 *μ*M, triclocarban and triclosan significantly inhibited human CYP19A1 to 5–9% of the control (Figure 4(a)). We further investigated the IC_50_ values of triclocarban and triclosan in the range of concentrations (10^−8^–10^−4^ *μ*M). IC_50_ values for triclocarban and triclosan were 15.81 and 6.26 *μ*M, respectively (Figure 5 and Table 1). This indicates that triclosan is more potent to inhibit CYP19A1 than triclocarban.

### 3.3. The Mode of Inhibition on Human CYP19A1

**Figure 6:**
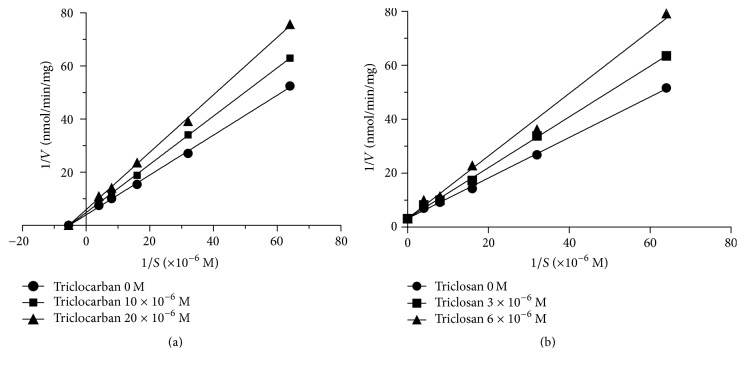
Inhibitory modes of triclocarban and triclosan on human CYP19A1 activity. Lineweaver-Burk plot in the presence of testosterone for different concentrations of triclocarban (10–20 *μ*M) and triclosan (3–6 *μ*M). *V* = pmol estradiol/mg protein·min. [*S*] = concentrations of testosterone. (a) Triclocarban; (b) triclosan.

The modes of triclocarban and triclosan in inhibition of human CYP19A1 activity were analyzed by enzyme kinetics (Table 1). When different concentrations of testosterone (15.6–250.0 nM) were used, Lineweaver-Burk plot demonstrated that triclocarban (Figure 6(a)) was a noncompetitive inhibitor, while triclosan (Figure 6(b)) was a competitive inhibitor of CYP19A1.

### 3.4. Docking of Triclocarban and Triclosan to CYP19A1

**Figure 7:**
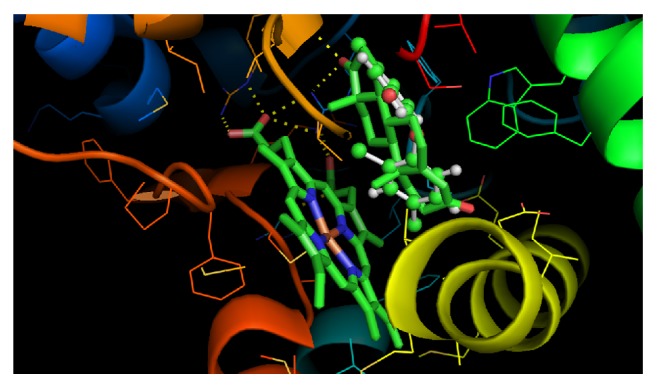
Docking of triclosan to human CYP19A1. The crystal structure of recombinant human CYP19A1 (PDB ID: 4KQ8) with heme and androstenedione was docked. It shows the binding of triclosan to the steroid-binding site of CYP19A1 similar to testosterone. Left green is heme structure; right green is testosterone structure; and the sky blue is triclosan, which exactly matches the testosterone-binding site.

Because the crystal structure of human CYP19A1 is available, we docked androstenedione into CYP19A1 first. The androstenedione was found to bind to the androstenedione-binding pocket with a hydrogen bond to MET374 residue (*A* = 1.698) of CYP19A1, with free energy of −9.13 Kcal. Docking analysis using triclocarban and triclosan (Figure 7 and Table 1) demonstrates that triclocarban does not bind to either heme or steroid-binding pocket, while triclosan binds to the steroid-binding pocket (Figure 7).

The free energies of triclocarban and triclosan are −6.899 and −7.397 Kcal, respectively. These data indicate that triclosan has higher binding affinity than triclocarban.

## 4. Discussion

In this study, we tested the inhibitory potencies of triclocarban and triclosan on human CYP19A1 activities. The suppression of CYP19A1 leads to the reduction of DHEA-mediated estradiol production.

Triclocarban and triclosan have different potencies to suppress human CYP19A1. The higher potency was found for triclosan (IC_50_: 6.26 *μ*M), which is 2.5 times higher than triclocarban (IC_50_: 15.81 *μ*M). Free energies of triclocarban and triclosan are higher than that of androstenedione (−9.125 Kcal), indicating that their affinity are lower than that of androstenedione. When comparison of free energy was performed for triclocarban and triclosan, we found that triclosan has a lower free energy (−7.397 Kcal), while triclocarban has a higher free energy (−6.899 Kcal), indicating that triclosan has higher affinity to bind to human CYP19A1 than triclocarban.

Triclocarban and triclosan have 2.5-fold differences regarding their potencies to inhibit human CYP19A1. This could result from the difference in their chemical structure. Triclosan has a phenol structure, being much similar to estradiol or other phenols such as bisphenol A. Indeed, the docking study using human CYP19A1 showed that triclosan binds to the testosterone binding pocket (Figure 7). In this regard, triclosan could bind to the steroid active sites to competitively inhibit CYP19A1 activity. The determination of mode of inhibition for triclosan indeed shows that it competitively inhibits CYP19A1.

If we compare the structures of triclocarban and triclosan, we will find that they have similar chloro groups. However, docking study does not show that triclocarban can bind to the steroid-binding pocket, indicating another mechanism for the inhibition of CYP19A1. Indeed, triclocarban noncompetitively inhibited human CYP19A1 (Figure 6(a)).

The inhibition of CYP19A1 by triclocarban and triclosan indeed caused the lower production of DHEA-mediated estradiol in JEG-3 cells (Figure 3), confirming that these three chemicals also penetrate the cell membrane to get into the cells to act. JEG-3 cells could produce human chorionic gonadotropin, a steroid-stimulator hormone, at 27 ± 3 (mean ± SEM) ng/mg cellular protein × 24 h and progesterone (22 ± 2.5 ng/mg cellular protein × 24 h) but could produce estrogen steroids. Therefore, the addition of DHEA was required to produce estrogen [14]. Indeed, the serum estradiol levels of pregnant rats that received 600 mg/kg/day triclosan from gestational day (GD) 6 to 20 were significantly reduced [8]. Triclocarban also inhibited the brain-specific expression of aromatase in early zebrafish embryos [21]. Thus, both triclocarban and triclosan could interfere with estradiol production.

In this study, we demonstrated that triclocarban and triclosan could inhibit estradiol production in JEG-3 cells around 100 nM. Nevertheless, these observations are relevant to public health, because triclocarban and triclosan occur in the environment at comparable levels. It has been reported that the levels of triclocarban and triclosan in human urine samples were significantly higher [4, 5]. Such concentrations of triclosan and triclocarban have the potential to inhibit CYP19A1 activity.

In conclusion, the present study demonstrated the inhibitory potencies of triclosan and triclocarban on human CYP19A1. It should be noted that triclosan is a potent inhibitor of human CYP19A1, thus leading to a possible reduction of estradiol.

## Figures and Tables

**Table 1:** The enzyme kinetics parameters and the half maximal inhibitory concentration (IC_50_) and modes of action of triclocarban and triclosan.

	Triclocarban	Triclosan
IC_50_ (×10^−6^ M)	15.807 ± 0.110	6.265 ± 0.102
Mode versus testosterone	Noncompetitive	Competitive
Free energy: androstenedione	−9.13	−9.13
Free energy: chemical	−6.899	−7.397
Binding	Not in the steroid site	In the steroid site

Mean ± SEM, *n* = 4.
